# On Heidis, Howards, and Hierarchies: Gender Gap in Medicine

**DOI:** 10.3389/fped.2022.901297

**Published:** 2022-04-26

**Authors:** Michele Kong

**Affiliations:** Department of Pediatrics, The University of Alabama at Birmingham, Birmingham, AL, United States

**Keywords:** women, gender, inequality, gap, diversity, inclusion

As a young child, I was always known as a *tomboy*. The Merriam-Webster dictionary defines *tomboy* as a girl who behaves in a manner usually considered boyish. I loved climbing trees, scaling everything in sight, and playing with dirt, and I was never afraid to be just me. Growing up in a small fishing village, I was fortunate to be surrounded by mountains, trees, and beaches that formed the perfect landscape for my adventures. Aunties often gave me disapproving looks as I did not meet their standards of a quiet, obedient girl who was seen but not heard. I was expected to always smile and be perfect. Scrapes and bruises were seen as a reflection of my disobedience; I was supposed to learn how to cook and sew, not take risks with the boys as they jumped headfirst into the water and off cliffs. These unforgiving expectations continued into adulthood, made worse by a society that was biased against women.

While gender inequality can be seen across economic, social, political, intellectual, and numerous other domains, my experience in medicine made me realize that unless we act singularly and collectively, the gap will only continue to widen for our sisters and daughters. Even though more than 50% of medical students in the United States are female^1^, gender parity is still not reflected in academia and medical leadership. When female physicians enter the workforce, they find themselves with lower compensations and less institutional support compared to their male counterparts, even when their productivity is higher or comparable. The disparity becomes even more striking in top executive positions that are mission critical such as department chairs, deans, presidents, or even hospital CEOs.

In a recent study involving ~1.3 million patients, female patients were less likely to experience complications and to die if treated by a female rather than a male surgeon (1). Investigators also found that patients treated by female internists had significantly lower mortality and readmission rates compared with those cared for by male internists within the same hospital, even after adjusting for potential confounders (2). Likewise, numerous other studies have also demonstrated that women physicians tend to be more collaborative and are stronger communicators than their male colleagues–attributes that can lead to improved patient outcomes and satisfaction. Yet, the workplace remains unconducive for the retention and advancement of women. For the same position, the administrative work burden is often higher for women, with a pay gap seen between women and men physicians within all medical specialties. On a micro level, gender biases also continue to prevail, whether conscious or otherwise. I can recount the numerous times when I was introduced by my first name while male peers were introduced within the same setting by their titles. In other instances, the assumption was made that I was the trainee or nurse, and the male trainee was assumed to be the attending or physician in charge, when in fact it was exactly the opposite. I would have comments made regarding my Asian features and youthfulness, minimizing the intellect and skills that I brought to the table. This seems especially true in more technical disciplines, such as surgery or critical care when people tend to view women who look feminine as less competent.

As a child, and into adulthood, I found myself wired such that I was always leaning in, always competing to accomplish a goal, to be the best, and going the distance to prove myself. In my drive to excel, I have been called intense and assertive, in ways that did not always come across as compliments. In a classic case study by Professor Frank Flynn at Columbia Business School, we saw how changing the name of a successful Silicon Valley venture capitalist, Heidi Roizen to Howard Roizen, impacted how she was judged as a person^3^. Although students rated Howard and Heidi as equally competent, they much preferred Howard and liked him significantly more. Heidi was thought to be selfish and too aggressive, with few wanting to work for or hire her. On the other hand, Howard was regarded as someone that they would want to hang out with, who would make a great colleague and even as a boss. Gender bias penalizes women for behaving in a certain way, especially when it violates what society regards as gender norms. The dominant, assertive, and authoritative behaviors that we associate with leadership tend not to be viewed as attractive in women. Rather, women are supposed to be kind, friendly, deferential, and socially skilled. The incongruity between our biases on gender and leader stereotypes inherently leads to a prejudice where our female leaders are judged more harshly even when they outperform their male counterparts.

If we do not act now, we will continue to have an exodus of female physicians from our profession. We are losing them in the workforce and missing out on having them in leadership positions. We need to disrupt gender stereotypes and insist on systemic and cultural change that will make gender equity and inclusion more than just words. In our day to day, we need to speak up when we witness gender bias. We need to push against comments that are made about our looks and personality, emphasizing, rather our intellect and skillset. We need to stand up for each other and be the other women's sponsors. It is important to share opportunities and always invite other women to the table. We need to be vocal and champion their recognition and advancement. Perhaps it is as simple as taking the opportunity when a woman voices her opinion in a meeting to acknowledge her contribution specifically and restate the point made. We need to amplify the other woman. By taking these small yet tangible steps, we can begin to effect change where we are at this very moment. For those who are in leadership positions, we need to be not just mentors but sponsors of other women. This means using political influence and personal clout to advocate for and place a more junior person in a key position.

Gender inequality is hurting us all as a society. Unless we have true equity, diversity, and inclusion in our workplace, leadership, and culture, we will never achieve the best version of our world. This is a gap that we cannot afford to ignore.

## Author Contributions

The author confirms being the sole contributor of this work and has approved it for publication.

## Conflict of Interest

The author declares that the research was conducted in the absence of any commercial or financial relationships that could be construed as a potential conflict of interest.

## Publisher's Note

All claims expressed in this article are solely those of the authors and do not necessarily represent those of their affiliated organizations, or those of the publisher, the editors and the reviewers. Any product that may be evaluated in this article, or claim that may be made by its manufacturer, is not guaranteed or endorsed by the publisher.

## Footnotes

^1^Available online at: https://www.aamc.org/news-insights/press-releases/majority-us-medical-students-are-women-new-data-show (accessed March 18, 2022).

^2^Available online at: https://www.gsb.stanford.edu/experience/news-history/gender-related-material-new-core-curriculum (accessed March 18, 2022).
